# How experts on autoinflammatory diseases classify inherited periodic fevers: preliminary results of the Eurofever Delphi Survey

**DOI:** 10.1186/1546-0096-13-S1-P146

**Published:** 2015-09-28

**Authors:** S Federici, F Vanoni, S Ozen, J Frenkel, H Lachmann, A Martini, N Ruperto, M Hofer, M Gattorno

**Affiliations:** 1 Pediatria II - Reumatologia, Istituto G. Gaslini, Genova, Italy; 2 CHUV, Pediatric Rheumatology Unit, Lausanne, Switzerland; 3 Department of Pediatric Nephrology and Rheumatology, Hacettepe University, Ankara, Turkey; 4 Department of Paediatrics, University Medical Center Utrecht, Utrecht, Netherlands; 5 National Amyloidosis Centre, Royal Free Campus, University College Medical School, London, UK

## Background

Provisional evidence-based classification criteria for Familial Mediterranean Fever (FMF), Cryopyrin Associated Periodic Syndrome (CAPS), Tumor Necrosis factor Receptor Associated Periodic Syndrome (TRAPS) and Mevalonate Kinase Deficiency (MKD) have been recently developed based on data coming from the Eurofever registry. However, no consensus on how to combine clinical criteria with results of molecular analysis has been reached so far.

## Objectives

To understand how physicians involved in the clinical care of patients with Autoinflammatory diseases (AIDs) classify patients with inherited periodic fever in daily practice.

## Methods

By using the Delphi and Nominal Group Technique, we started a process made of three consecutive e-mail surveys. In the first survey, clinicians/biologists and other health professionals working in the field of autoinflammation were asked to identify the variables that they consider as important, in their clinical practice, for the diagnosis of patients with inherited periodic fever. This survey was open not to influence the experts.

## Results

We sent the first survey to 124 experts. The overall rate of response was 107 (86%): 101 experts responded to be interested in the survey and 88 completed and confirmed it for at least one disease; 6 experts responded not to be interested. No clinical variable was chosen by all the experts for any disease the five most cited clinical variables for FMF were recurrent fever (80% of experts), abdominal pain (67%), arthritis (53%), thoracic pain (47%) and arthralgia (36%). The five most cited clinical variables for CAPS were fever (75%), urticarial rash (71%), hearing loss (49%), ocular involvement (40%) and arthralgia (35%). The five most cited clinical variables for TRAPS were long lasting fever (92%), rash (84%), periorbital edema (59%), myalgia/myositis (57%), and abdominal pain (55%) while the five most cited clinical variables for MKD were abdominal pain (61%), fever (59%), skin rash (41%), diarrhea (43%) and arthralgia (39%). A confirmatory genetic test resulted a relevant element for the diagnosis of FMF, TRAPS, CAPS and MKD while the response to treatment for FMF and CAPS.

## Conclusions

The preliminary results of the first Eurofever Delphi Survey show a high rate of response by Expert, underlying the interest of the scientific community in this topic. A wide heterogeneity in their response was observed. At the end of the Delphi process, we will obtain different set of clinical criteria whose performance will be tested in comparison to already existing criteria in a cohort of patients affected by AIDs. The final step will be a Consensus among experts (geneticists and clinicians) in order to define the best combination of clinical and genetic data for the definitive classification of patients with inherited periodic fevers.

